# Anticoagulation and antiplatelet therapy in acute coronary syndromes: choosing between the Scylla of bleeding and the Charybdis of ischaemic events

**DOI:** 10.1007/s12471-018-1121-5

**Published:** 2018-05-24

**Authors:** R. J. de Winter

**Affiliations:** Department of Cardiology, Academic Medical Center, Amsterdam, The Netherlands

Homer described the mythical sea monsters Scylla and Charybdis, situated close together on opposite sides of the Strait of Messina between the mainland of Italy and the island of Sicily. When Odysseus was forced to choose between the risk of crashing on the rocky heads of Scylla or losing the entire ship in the whirlpool of Charybdis, he opted to pass by Scylla and loose only a few sailors [1]. Likewise, as cardiologists caring for our patients, we often choose to accept the Scylla of a few bleeding events, preferring to avoid the Charybdis of cardiovascular death or myocardial infarction. We fear Charybdis because we have seen it, we take our chances with Scylla because bleeding events often occur outside our field of vision.

In this issue of the Netherlands Heart Journal, under the editorship of J. ten Berg, the authors review the evidence on which the current guidelines are based and describe how we may guide our clinical decision making. And let’s acknowledge right here and now: it has become quite complicated! As we try to decide on the best treatment options for our patients, we deal with three clinical domains: acute coronary syndromes, percutaneous coronary interventions and atrial fibrillation. In a recent review summarising the selection of P2Y12 inhibitor treatment in patients with acute coronary syndrome, Tantry et al. show that recommendations come from guidelines on percutaneous coronary intervention, a guideline focused update on duration of dual antiplatelet therapy in patients with coronary artery disease, guidelines on the management of patients with non-ST-elevation acute coronary syndrome (NSTE-ACS), and guidelines on the management of patients with ST-elevation myocardial infarction (STEMI) [2]. If we factor in the presence of atrial fibrillation, a condition that will become more and more frequent in our aging patient population, we can add several more guidelines. Deciding on anticoagulation and antiplatelet therapy and their combination in the treatment of ACS patients with atrial fibrillation that have undergone coronary stent placement, we have a choice of two vitamin K antagonists (acenocoumarol, fenprocoumon), four non-vitamin K oral anticoagulants (dabigatron, apixaban, rivaroxaban, edoxaban) and five antiplatelet agents (aspirin, clopidogrel, prasugrel, ticagrelor, cangrelor). Then we must decide on the duration of the prescribed medication, 1 month, 3 months, 6 months, 12 months, or beyond 12 months, which altogether leaves us faced with a mind-boggling 100 possible combinations.

Needless to say, this is a “moving target” with trial results becoming available every few months, leaving physicians in search of guidance for everyday clinical decision making. One important development in this respect is the availability of new-generation drug-eluting stents that have a much lower risk of stent thrombosis, both in stable patients and in patients with acute coronary syndromes, in the short-term as well as the longer follow-up. This has, at least in part, changed the risk-benefit balance of longer duration dual antiplatelet therapy (DAPT) beyond 12 months as the prevention of stent thrombosis may be less relevant. In a recent review, Bittl et al. [3] conclude that evidence from randomised controlled trials suggest that patients undergoing implantation with safer, new-generation drug-eluting stents may be treated with a minimum of 3 to 6 months of DAPT. Moreover, they conclude that “The declining risk of late stent thrombosis with newer-generation DES and the inability to predict life-threatening bleeding limit the appeal of 18 to 48 months of DAPT over 6 to 12 months of therapy. In contrast, patients with prior MI at high risk of atherothrombosis experience fewer ischemic events with prolonged DAPT at a cost of increased bleeding events”. Shorter DAPT is an increasing trend. Palmerini et al. [4] showed in a recent network meta-analysis that “short DAPT was associated with lower rates of major bleeding compared with 1‑year DAPT, irrespective of clinical presentation. All-cause mortality was not significantly different with short versus long DAPT in patients with stable CAD and in patients with ACS”. In contrast, Parker and Storey [5] conclude, based on the results of the PEGASUS TIMI 54 trial, that “high-risk patients with a history of MI gain benefit from a longer duration of ticagrelor-based DAPT”. It is clear that we need to consider ischaemic and bleeding risks in individual patients when deciding on the duration of therapy and this is recognised in the current NSTE-ACS guidelines of the European Society of Cardiology.

This issue will provide guidance. Hermanides et al. [6] describe optimal pharmacotherapy in STEMI patients, van Kuijk et al. [7] summarise treatment options in NSTE-ACS and Kikkert et al. [8] give a comprehensive overview of the evidence for shorter, longer or “optimal” DAPT duration in ACS. Pisters et al. [9] guide you in the antithrombotic management of patients with atrial fibrillation and van Vugt et al. [10] provide the data on the stricter recommendations on anticoagulation around electrical cardioversion for atrial fibrillation. Gimbel et al. [11] describe useful treatment protocols for discontinuing and restarting anticoagulation and antiplatelet treatment when a bleeding event has occurred. In their clinical itinerary, patients with ACS travel a tortuous path from non-PCI centre to PCI centre and back, from critical care unit to cardiology ward to outpatient post ACS clinic, to cardiac rehabilitation and general practitioner. With this issue in hand, local protocols may be reviewed, updated if needed, and shared between all involved in the care of ACS patients.
